# Therapeutic drug monitoring for cytotoxic anticancer drugs: Principles and evidence-based practices

**DOI:** 10.3389/fonc.2022.1015200

**Published:** 2022-12-08

**Authors:** Pattanaik Smita, Patil Amol Narayan, Kumaravel J, Prakash Gaurav

**Affiliations:** ^1^ Department of Pharmacology, Post Graduate Institute of Medical Education and Research, Chandigarh, India; ^2^ Department of Clinical Hematology and Medical Oncology, Post Graduate Institute of Medical Education and Research, Chandigarh, India

**Keywords:** therapeutic drug monitoring, cytotoxic drugs reconstitution, busulfan, methotrexate, 5-FU, 5 fluorouracil, precision medicine

## Abstract

Cytotoxic drugs are highly efficacious and also have low therapeutic index. A great degree of caution needs to be exercised in their usage. To optimize the efficacy these drugs need to be given at maximum tolerated dose which leads to significant amount of toxicity to the patient. The fine balance between efficacy and safety is the key to the success of cytotoxic chemotherapeutics. However, it is possibly more rewarding to obtain that balance for this class drugs as the frequency of drug related toxicities are higher compared to the other therapeutic class and are potentially life threatening and may cause prolonged morbidity. Significant efforts have been invested in last three to four decades in therapeutic drug monitoring (TDM) research to understand the relationship between the drug concentration and the response achieved for therapeutic efficacy as well as drug toxicity for cytotoxic drugs. TDM evolved over this period and the evidence gathered favored its routine use for certain drugs. Since, TDM is an expensive endeavor both from economic and logistic point of view, to justify its use it is necessary to demonstrate that the implementation leads to perceivable improvement in the patient outcomes. It is indeed challenging to prove the utility of TDM in randomized controlled trials and at times may be nearly impossible to generate such data in view of the obvious findings and concern of compromising patient safety. Therefore, good quality data from well-designed observational study do add immense value to the scientific knowledge base, when they are examined in totality, despite the heterogeneity amongst them. This article compiles the summary of the evidence and the best practices for TDM for the three cytotoxic drug, busulfan, 5-FU and methotrexate. Traditional use of TDM or drug concentration data for dose modification has been witnessing a sea change and model informed precision dosing is the future of cytotoxic drug therapeutic management.

## Introduction

**Figure 1:**
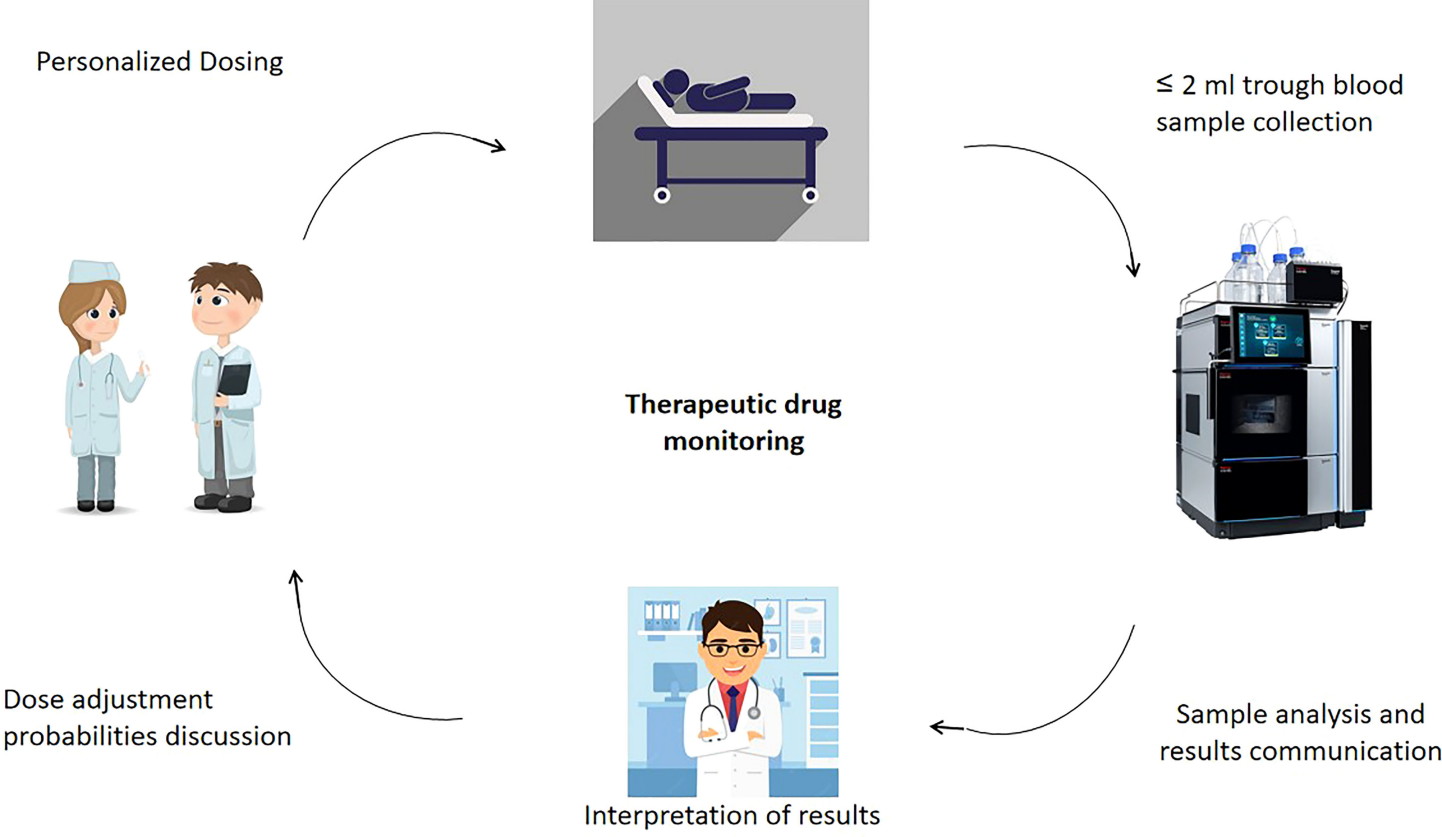
Overview of TDM applications in cytotoxic anticancer drug therapy.

Cytotoxic drugs are the oldest class of anticancer drugs. They are highly efficacious and also have low therapeutic index; therefore, a great degree of caution needs to be exercised in their usage. To optimize the efficacy, these drugs need to be given at the maximum tolerated dose. This dose leads to a significant amount of toxicity to the patient. The fine balance between efficacy and safety is the key to the success of cytotoxic chemotherapeutics. However, it is possibly more rewarding to obtain that balance for this class drugs as the frequency of drug-related toxicities is higher compared to the other therapeutic class and is potentially life-threatening and may cause prolonged morbidity. Significant efforts have been invested in the last three to four decades in therapeutic drug monitoring (1) research to understand the relationship between the drug concentration and the response achieved for therapeutic efficacy as well as drug toxicity for cytotoxic drugs. TDM evolved over this period and the evidence gathered favored its routine use for certain drugs. Since TDM is an expensive endeavor, both from an economic and a logistic point of view, to justify its use, it is necessary to demonstrate that the implementation leads to perceivable improvement in patient outcomes. It is indeed challenging to prove the utility of TDM in randomized controlled trials and at times may be nearly impossible to generate such data in view of the obvious findings and concern of compromising patient safety. Therefore, good quality data from a well-designed observational study add immense value to the scientific knowledge base, when they are examined in totality, despite the heterogeneity among them. This article compiles the summary of the evidence and the best practices for TDM for the three cytotoxic drug, busulfan, 5-FU, and methotrexate (Mtx) (**Figure 1**).

This article compiles the summary of the evidence for three cytotoxic drugs, busulfan, 5-FU, and Mtx, where the case for TDM is much established. Though there is evolving evidence for TDM in favor of the platinum group of drugs (paclitaxel, docetaxel, and carboplatin), tyrosine kinase inhibitors (1), and others like abiraterone and tamoxifen, they are beyond the scope of this review as we primarily intend to bring out the TDM best practices for the established cytotoxic drugs only (1–4).

## Busulfan

Busulfan is a bifunctional DNA alkylating anticancer agent and acts in a cell cycle nonspecific manner. After systemic absorption, the carbonium ions are rapidly formed, which react with guanine molecules of the DNA through a nucleophilic substitution reaction (SN2) (5) forming intra- and interstrand crosslinks. This leads to breaks in the DNA molecule as well as crosslinking of the twin strands, resulting in interference of DNA replication and transcription of RNA and hence cell proliferation (6, 7).

### Clinical use of busulfan

Oral busulfan had been used for the treatment of chronic myeloid leukemia (CML) (8, 9) and other myeloproliferative disorders since the 1950s due to the inhibitory activity of the sulfonic derivatives of the drug on hematopoiesis (10, 11). In the last few decades, this use has become less popular due to the unsatisfactory curative potential despite initial good cytoreduction, thus reducing its use as a palliative therapy only. It was also found to have dismal efficacy in certain subsets of patients like those who have Philadelphia negative CML (12). In the present time, the use of oral busulfan is limited to palliative treatment of CML (myeloid, myelocytic, and granulocytic) and myeloproliferative neoplasms (13). In the late 1980s, oral busulfan was started to be used as a pretransplant myeloablative agent along with cyclophosphamide (8, 14). Oral administration of busulfan results in significant gastrointestinal irritability leading to nausea and vomiting that typically led to unpredictable systemic bioavailability (15, 16). Erratic absorption from the intestine coupled with GI complications substantially increased the systemic exposure variability to more than 10-fold (reported bioavailability ranged from 20% to 99%) (9). This unpredictability was responsible for poor efficacy and higher failure rate of the oral regimen and therefore paved the way for a major refinement in busulfan delivery. The i.v. formulations of busulfan were developed by Anderson and colleagues at the MD Anderson Cancer Center in the USA and was approved by the US FDA in 1999 (17, 18). Later on, intravenous busulfan replaced the oral route, as it provided better control over drug administration, bringing down the intradose exposure variation to 2- to 2.5-fold and maximizing the antitumor efficacy (19).

Though the use of oral busulfan was short-lived and it has now been replaced by intravenous use, it provided great insight to understand the pharmacokinetics (PK) of the drug and helped in the evolution of the TDM strategy. The PK profile of the drug is best described as a single compartment model, with rapid absorption and maximum plasma concentration achieved within 1 h. Majority of the drug is metabolized in the liver by the glutathione-S-transferase (20) enzyme and the metabolites are excreted *via* urine. A minimal amount of the drug (<2%) is excreted unchanged in the urine. The drug is eliminated by a log-linear fashion. We will discuss the PK of i.v. administered drug especially in the context of high-dose busulfan therapy as a part of myeloablative regimen before hematopoietic stem cell transplantation (HSCT). It is generally administered at a dose of 0.8 mg/kg in normal saline or 5% dextrose 6 hourly infusion over 2 h (or 3.2 mg/kg daily). Sixteen such doses (or four doses for once daily doses) are administered over 4 days (−7, −6, −5, and −4 days when day 0 is the day for infusion of the hematopoietic stem cell transfusion). The PK profile of the i.v. drug is similar to that of oral administration, which skips the fast-pass metabolism, providing 100% bioavailability.

High-dose intravenous busulfan has now been the preferred choice for myeloablation at most of the HSCT centers compared to total body irradiation (TBI) (12). It is currently the standard of care in the pretransplant myeloablative conditioning regimens along with other lymphotoxic chemotherapeutic agents (cyclophosphamide and fludarabine), for hematopoietic stem cell transplantation (HSCT). HSCT is a widely used potentially curative treatment strategy for several diseases, including hematological malignancies (e.g., leukemia and lymphoma), solid tumors, and nonmalignant disorders (e.g., thalassemia and sickle cell anemia).

### Justification for TDM of busulfan

As described above, though busulfan is a highly efficacious cytotoxic drug, it poses several challenges in optimum dosing. Many factors qualify busulfan as an ideal candidate for TDM. The need for optimization of the dose was realized right at the beginning of the therapeutic failure of the oral regimen due to inadequate exposure. On the other hand, the serious adverse drug effects associated with higher exposure of busulfan lead to both hematological and nonhematological events. The onset of some of the ADRs are acute and some lead to chronic effects due to exposure over longer periods (21). The most common ADR include acute graft versus host disease (GVHD), mucositis, myelosuppression, seizures, hepatic veno-occulsive disease (HVOD, also known as sinusoidal occlusion syndrome), bronchopulmonary dysplasia, pulmonary fibrosis, and embryo–fetal toxicity. Robust evidence from several clinical studies support direct relationship between exposure–efficacy–toxicity. The introduction of the i.v. formulation led to better control over the concentration of the drug achieved in the systemic circulation circumventing the unpredictable intestinal absorption and hepatic fast-pass metabolism. However, the need for TDM was realized because of the bimodal distribution in the posttransplant mortality. Those who achieved inadequate concentration or those who achieved undesirably high concentration succumbed. With 6 hourly regimen, the threshold for therapeutic response is an exposure over one dosing interval, which is found to be 900–950 μM·min, whereas the threshold for toxic adverse effects is 1,200–1,500 μM·min. Hence, it can be concluded that busulfan has a narrow therapeutic window (900–1,200 mM·min) and consequently has a low therapeutic index (1,500/900 is <2).

With the use of i.v. formulation, the systemic concentration of busulfan had attained better predictability. Previously, interindividual variability of busulfan bioavailability with oral administration varied more than 10 fold, whereas it reduced to 2- to 2.5-fold with the use of i.v. formulation (19, 21). The reported coefficient of variation in busulfan maximal plasma concentration, clearance, and AUC was 18%, 25%, and 25% (13, 18) (Otsuka America Pharmaceutical Inc., Rockville,). Several subsequent clinical studies also confirmed the same (19, 22). The globally reported interindividual variability with i.v. use of busulfan is about 30%. Moreover, the intraindividual variability in exposure for the same dose may be as high as fivefold. Pharmacogenetic alterations in the metabolizing enzymes, drug transporters, and chronopharmacology have been studied in this context. Single-nucleotide polymorphisms (SNPs) of the GST isoenzymes (GSTA1, GSTM1, and GSTP1) have been demonstrated to significantly affect the PK (clearance) of busulfan, thereby contributing to interindividual variability in small-sized studies and mostly in a pediatric population (23–25). However, their effect on the pharmacodynamics and incidence of therapeutic success vis-à-vis adverse events is not clear and hence their clinical relevance has not yet been demonstrated (26, 27). Similarly, polymorphic variants of CYP 2C9, 2B6, and membrane transporters like ABCB1 and SLC have also been studied for their association with busulfan disposition. Nevertheless, their role has not been found to be consequential (28). Chronopharmacologic variability in GST and CYP enzyme activity have also been studied in the context of variability in PK of busulfan. It has been postulated that this may contribute to intraindividual variability of AUC for the same dose of busulfan. However, these studies were conducted in the earlier period with oral busulfan and had several operational confounding factors, most importantly changes in intestinal motility. Moreover, the results from other studies have also yielded conflicting results (29, 30). To summarize, it was observed that the interindividual variability is significant enough with high-dose intravenous busulfan therapy to merit practice of TDM.

In addition to the above factors, potentially important drug–drug interactions may be kept in mind to optimize busulfan exposure and TDM could help in dose and exposure optimization. Several classes of drugs have been studied (**Table 1**). The most important class that needs a cautious approach is antifungal drugs, and the strategy of spacing the prescription is adopted at many centers to avoid the interaction. Another drug that merits mention is phenytoin, which had been the most common antiseizure prophylaxis prescribed during high-dose busulfan therapy, which potentially induces busulfan clearance. The newer antiepileptics like levetiracetam is used these days to avoid the interaction. Nevertheless, TDM can be a very useful tool in case of inadvertent drug interaction.

**Table 1:** Potential drug interactions with busulfan.

Drug class and agents	Interaction with oral busulfan	Interaction with i.v. busulfan
Antifungals: Itraconazole Fluconazole Voriconazole Posaconazole Isavuconazole	Likely Exercise caution	Likely Exercise caution and avoid starting these drugs during preconditioning
Antiepileptic: Phenytoin	Potential	Potential
Antimicrobials: Metronidazole Ciprofloxacin	Inconsequential for both	Inconsequential for both
Others: Deferasirox Acetaminophen N-acetylcystine Oral contraceptive pills	Potential Unlikely Unlikely Unlikely	Potential Unlikely Unlikely Unlikely

Other factors contributing to variability in busulfan exposure are age and body composition. Clearance of busulfan is 30% higher in children compared to adults, which has been postulated to be due to the higher physiological activity of the GST in young age groups. Patients with higher fat composition have a prolonged elimination half-life for busulfan as the fat tissue behaves as a temporary storage compartment for busulfan due to its high lipid solubility (31).

### Evidence for exposure–adverse event relationship

At the advent of the oral conditioning regimen by Santos et al., a classical veno-occlusive disease of the liver was reported in the early 1980s, and by the end of the 1980s, it was suggested that TDM could have a potential role in reducing such adverse events (32–36). The oral dose regimen underwent several modifications; however, it was increasingly realized that several adverse events other than HVOD were associated with higher exposure to busulfan like acute GVHD (a cytokine storm that damages the normal organs in the body), seizures (typically of generalized type), and total incidence of treatment-related mortality. Though a multivariate analysis confirmed that oral administration is the single most important factor for HVOD, several other concomitant drugs like cyclophosphamide used in the preconditioning regimen, individual patient susceptibility, and disease type were also found to contribute to the event (12). Nevertheless, HVOD and hepato-renal failures are considered to be a classical trademark toxicity of high-dose busulfan therapy (12). Grochow and Dix demonstrated that the hepatic events are related to the drug concentration (34, 37). Both the studies indicated that an AUC of >1,500 μM·min and a steady-state plasma concentration (C_ss_) above 1,025 ng/ml were significantly associated with HVOD after 6 hourly oral administration of busulfan. The evidence from these studies confirmed that the threshold to HVOD is about 1,500 μM·min for a 6 hourly dosing (22, 38–40). Other studies, which used single daily dose administration, showed that the incidence of death due to HVOD was significantly increased above the exposure level of 6,000 μM·min (41, 42), hence confirming a full concordance between the findings. Similarly, the incidence seizure occurrence during treatment were significantly more during the higher exposures (30, 43). This is clearly explained by the PK profile of the drug, busulfan being lipophilic, and small molecules crossing the blood–brain barrier, and a higher concentration is achieved in the brain (1.3:1.0), hence causing acute neurotoxic effects. Apart from reducing toxicity, a pooled analysis of data of 674 patients who underwent stem cell transplant has shown that maintaining busulfan concentration within a therapeutic range led to better posttransplant survival. In the study, event-free survival at 2 years was 77.0% in patients with an optimum intravenous busulfan AUC of 78–101 mg·h/L compared with 66.1% in patients at the low historical target of 58–86 mg·h/L. Moreover, acute toxicity (*p* = 0.011) and transplant-related mortality (*p* < 0.0001) were significantly higher in high (>101 mg·h/L) busulfan AUC (44).

### Evidence for exposure–efficacy relationship

A substantial number of studies in the early period during the oral busulfan use documented therapeutic failures. The patients with nonmalignant disease were especially prone to failed engraftment due to the intact immune system and faster clearance of busulfan. On the other hand, the patients with malignant disease failed engraftment, which led to the relapse and recurrence (45, 46) of the disease (47, 48). Slattery et al. suggested a concentration cutoff of 1,250 μM·min and a Css of 925 ng/ml for optimal therapeutic effect in bone marrow transplantation (39). Subsequently, they performed the study in patients with CML and showed that targeting an AUC of >1,350 μM·min led to treatment success (38). Radich et al. also showed similar findings; with AUC >1,350 μM·min, excellent outcome was observed (49). More recently, Anderson et al. showed that an AUC value <900 μM·min was associated with higher failure rates in the 6 hourly i.v. busulfan regimen (22). There was another interesting observation that confirmed the exposure–response relationship for busulfan. Children who clinically tolerated oral busulfan better than adults perhaps experienced more relapse after HSCT due to graft failure. As the understanding in busulfan PK was consolidated for its effect related to age, it was found that these children had faster clearance of busulfan and hence had reduced exposure to the drug, leading to better tolerance as well as therapeutic failure (48, 50, 51). Hence, the TDM-based dose adjustment to keep the AUC above 900 μM·min has been the consensus agreement for achieving desirable therapeutic effect. Several large retrospective studies established a therapeutic window for clinical response between 900 and 1,500 μM·min for AUC over 6 h in a 16-dose administration schedule. The corresponding cumulative AUC over 16 doses was set at 144,000 to 240,000 mM·min (32, 38, 39, 52, 53).

### Evolution of the estimate of choice for TDM

In the 1980s, it was realized that single time point concentration would not be enough due to erratic absorption. Hence, exposure needs to be determined. With intravenous dosing, the concept of monitoring steady-state concentration was floated. Researchers came up with several formulas to calculate the Css and extrapolate a Css curve with first-dose AUC assuming that an ideal true steady state is reached. However, this was not a universal phenomenon, and a variation of 500% is noted between days 1 and 4 of infusion, as the volume of distribution (Vd) appeared to change with time. The hepatic enzyme activity decreases with progressive administration; hence, higher concentrations are observed in pretreated patients. Therefore, targeting the Css was found to be difficult and only 55% could achieve it even with best possible dose titration. Subsequent studies demonstrated that the exposure over a single dose is a better parameter to target. Hence, a limited sampling approach is adopted and the exposure is assessed by the calculation of area under the curve over the dosing interval (AUC0-τ), which is the parameter of interest for TDM (54). The current best practices for sample collection is described in the subsequent section.

### Evidence for usefulness of TDM for busulfan

The published literature that constitutes the evidence base for deployment of TDM in routine patient care is very heterogeneous. Adequately powered controlled clinical studies are sparse. Despite this, the expert opinion fully favors practice of TDM for personalized busulfan therapy so as to optimize the exposure, maximize the therapeutic effect, and minimize the adverse events. This is applicable only in the setting of high-dose busulfan for preconditioning before HSCT but not reduced intensity preconditioning (55). The landmark randomized controlled study by Grochow et al. demonstrated a reduction of HVOD to reduce from 75% to 15% when the busulfan dose titration was done based on the TDM data to keep the AUC below 1,500 μM·min and only 5% of the patients were maintained within therapeutic range. Thereafter, it had been really difficult to refute this finding by conducting randomized controlled studies, as it involved concerns of patient safety. Several studies conducted in different settings (different age groups like adult and children; several disease conditions like CML, AML, and multiple myeloma; and different co-conditioning agents like cyclophosphamide and melphalan) encountered similar findings (5, 38, 41, 49, 56–59). Traditionally, oral busulfan was administered in 6 hourly doses to minimize the GI adverse events. The same schedule continued for i.v. drug also due to the short half-life of the drug. The subsequent phase I/II PK studies did not demonstrate that there is a significant difference in the busulfan volume of distribution, half-life, and clearance between 6 hourly and 24 hourly dosing (60). As expected, the C_max_ values achieved with once daily doses were higher compared to the 6 hourly doses, but it was found that the clinical outcomes like rates of disease relapse (61), overall survival (61, 62), and HVOD (61–63) were not significantly different. Though definitive conclusions were impossible to arrive at, due to significant heterogeneity between the studies with regard to the disease population, concomitant medications, and inconstant use of TDM, QID dosing and OD dosing are in general considered to be equivalent.

### Available assays for TDM of busulfan

A number of analytical techniques have been used for the quantification of busulfan (e.g., HPLC, LC/MS, and GC/MS) to measure circulating plasma concentrations. Liquid chromatography methods coupled with mass spectrometry have been the most commonly used technique for clinical care because of their sensitivity, specificity, and accuracy of results (64, 65). However, it is technically demanding and the sample processing and analysis may be time-consuming. Busulfan degrades quickly at room temperature; hence, the samples have to be analyzed quickly. It is also desirable to have a short turnaround time as the dose modification decisions have to be provided within a window of as less as 6 h. For delivering an efficient TDM service, these factors must be taken into consideration. Recently, several automated assays have been developed. Nanoparticle- and microparticle-based immunoassays are the two significant additions. These assays can be quickly performed without any sample processing steps. It has also been shown that they are in good conformity with the LC-MS/MS methods (20, 66) and are gaining acceptance.

### Best practice for TDM of busulfan

The steady-state plasma concentration for busulfan is not achieved over 6–24 h even if the half-life is 2–3 h. Therefore, several samples are needed to determine the exposure to the drug over a dosing interval. A limited sampling approach is adopted, and the best schedule for sample collection as per the FDA monograph for the first dose is 2 h (end of infusion), 4 h, and 6 h (just before the start of next infusion) after the start of the infusion. For subsequent doses another sampling time point (baseline, before the start of the infusion) is added. The blood sample should be collected in heparinized vials and placed on wet ice till centrifugation. The plasma should be separated within 1 h and transferred to cryo vials. All plasma samples should be frozen at −20°C until analysis. The samples are stable for 3–4 days at 2–8°C and up to 6 months at −20°C. The AUC over the dosing interval is calculated by the simple trapezoidal rule. For once daily dosing, the terminal part of the elimination curve is calculated from a preinfusion “0” concentration for the first dose or the preinfusion concentration for the next dose. AUC_0–24 h_ is calculated by adding AUC_0-6 h_ and AUC_6–24 h_. The FDA-recommended AUC and Css values are similar to the consensus statement by the American Society for Bone Marrow Transplantation Practice Guideline Committee. The AUC and Css targets for high-dose busulfan therapy is enumerated in **Table 2**. The attempt to optimize the exposure is started as early as the first day dose modifications.

**Table 2:** Summary of dose and exposure–concentration targets for high-dose busulfan therapy.

Pediatric population	Adult population
EMEA: Weight-based dosing* 4 days <9 kg:1.0 mg/kg Q6H or 4 mg/kg OD >9–<16 kg: 1.2 mg/kg Q6H or 4.8 mg/kg OD 16–23 kg: 1.1 mg/kg Q6H or 4.4 mg/kg OD 23–34 kg: 1.0 mg/kg Q6H or 4 mg/kg OD >34 kg: 0.8 mg/kg Q6H or 3.2 mg/kg OD FDA: Weight-based dosing* 4 days <12 kg: 1.1 mg/kg Q6H or 4 mg/kg OD >12 kg: 0.8 mg/kg Q6H or 3.2 mg/kg OD	EMEA and FDA dose recommendations are the same for adults for dosing as well as targets in adults 1.0 mg/kg Q6H * 4 days 4 mg/kg OD * 4 days
EMEA Target for 6 h dosing AUC: 1,125 (900–1,500) μM·min Css: 770 (650–1,026) ng/ml Target for 24-h dosing AUC: 4,500 (5,262–6,000) μM·min Css: 770 (650–1,026) ng/ml FDA Target for Q6H dosing AUC: 1,125 (900–1350 ± 5%) μM·min Css target: 770 (650–924) ng/ml Target for 24-h dosing AUC: 5,262 μM·min Css target: 900 ng/ml	EMEA and FDA Target for 6-h dosing AUC: 1,125 (900–1,500) μM·min Css: 770 (800–1,000) ng/ml Target for 24-h dosing AUC: 4,500 (5,262–6,000) μM·min Css: 900 (800–1,000) ng/ml

Dosing should be according to the ideal body weight or lean body weight, whichever is lower.

### Clinical impact of busulfan precision therapy strategies

Model informed precision dosing (MPID) has been gaining popularity over the last decade. These methods are attractive as they use less number of samples to predict the AUC and suggest dose modification using Bayesian forecasting. The model-informed dosing also takes into account the body size, age, organ function, etc. for dose prediction unlike the traditional dosing, which is weight based. Examples of some of the available software are insightRX and NextDose. The web-based services are www.insight-rx.com and https://doseme-rx.com. It has been shown to have relatively less bias in AUC estimations of busulfan compared to the conventional trapezoidal method (78, 79) and may be less prone for error that may creep in due to inadequate documentation of sampling time (80). The MIPD strategy may be software or web-based platforms.

## 5 Fluorouracil

5-fluorouracil (5-FU) is a pyrimidine analogue used in the treatment of several solid tumors (breast, colorectal, stomach, head, and neck). It is typically administered intravenously as prolonged infusion due to its very short half-life (20 min) (81).

After administration, 5-FU penetrates cells by a facilitated transport route, where it is transformed into fluorodeoxyuridine monophosphate (FdUMP). FdUMP inhibits the synthesis of the deoxythymidine monophosphate (dTMP) by forming complexes with the enzyme thymidylate synthase (TS). dTMP is integral to DNA replication and repair, and its depletion causes an imbalance of intracellular nucleotides, which allows the enzyme endonuclease to cause double-stranded breaks in the DNA (82).

When used orally, 5-FU shows poor absorption in the gastrointestinal tract. To maximize systemic absorption, parenteral administration of 5-FU is used when treating visceral cancers. 5-FU can be given intravenously as a bolus infusion over a period of days or as a “protracted” infusion using an ambulatory pump for 1 to 2 weeks. Fluorouracil is distributed throughout the body, including the liver, brain, bone marrow, CSF, and intestinal mucosa. About 80% of 5-FU is metabolized in the liver by dihydropyrimidine dehydrogenase (DPD) to the inactive metabolite dihydrofluorouracil (5-FUH_2_) (83). Due to the fast catabolism in the liver, the terminal half-life of 5-FU when administered intravenously is about 8 to 20 min. This finding can be explained by the saturation of 5-FU metabolism by DPD as plasma concentrations approach the Km of DPD, which is known to be approximately 4.6 mg/L, which subsequently causes a more than proportionate increase in 5-FU plasma concentrations with dosage (73). 5-FU undergoes dose-dependent elimination and is excreted *via* urine as unchanged drug within 6 h of 5% to 20% and metabolites over 3 to 4 h.

Diarrhea was the most often reported side effect in patients undergoing systemic 5-FU treatment. Dehydration, nausea, and vomiting are typical side effects. Neutropenia, pyrexia, pulmonary embolism, thrombocytopenia, and leukopenia are more serious side effects that need to be monitored in individuals receiving systemic 5-FU chemotherapy (82).

### Rationale for TDM of 5-FU

5-FU therapy meets the most important criteria for TDM, i.e., an established exposure–toxicity and exposure–clinical activity relationship. Currently, body surface area (BSA)-based calculation is used for dosing the fluoropyrimidine 5-FU (84). At typical doses, 5-FU’s therapeutic effectiveness is moderate, as the dosing is often limited by the safety profile, with myelosuppression and gastrointestinal toxicity being the most common side effects. The conservative dose adjustment steps without TDM support result in delayed nonattainment of requisite exposure and, therefore, lead to suboptimal therapeutic success. The exposure–toxicity and exposure–clinical activity relationship has been reported in several clinical studies both prospective and retrospective (**Table 3**).

**Table 3:** Evidence base in support of exposure–response–toxicity for 5-FU.

Study details	Disease	Pk parameter	Exposure–toxicity	Exposure–clinical response
Hillcoat (1978) (67) (original study)	GI malignancies	AUC	Not reported	RR with TDM: 40% RR without TDM: 5%
Thyss (1986) (68) (original study)	Head and neck cancer	AUC	Strong relationship	Good response rate
van Groeningen (1988) (69) (original study)	Advanced malignancies	AUC	Strong relationship	Not Available
Fety (1998) (70) (original study)	Head and neck cancer	AUC	Reduced adverse events	Good response rate RR with TDM: 18% RR without TDM: 8%
Gamelin (2008) (71) (original study)	mCRC	AUC	Strong relationship	Not Available
Yoshida (2008) (72) (original study)	mCRC	AUC	Strong relationship	Not Available
Wilhelm (2016) (73) (original study)	mCRC	AUC	Decreased toxicities	RR with TDM: 20% RR without TDM: 12%
Yang (2016) (74) (original study)	Colorectal cancer	AUC		Superior overall response rate
Fang (2016) (75) (original study)	Solid tumors	AUC	Lower the probability of grade 3/4 adverse drug events	Superior overall response rate
Salamone (2017) (76) (original study)	mCRC	AUC	Less rates of grade 3/4 adverse events	Good response rate
Deng (2020) (77) (original study)	mCRC	AUC	Incidence of adverse events reduced	Long-term efficacy improved

This is a nonexhaustive list, including only the important, landmark studies that led to the consensus for adoption of 5-FU TDM.

The second factor that adds to the justification for TDM is extremely high interindividual variability and intraindividual variability. While it was brainstormed in several professional meetings of physicians and laboratory scientists that there might be methodological variability in studies reporting 5-FU blood concentration, it would be important to evaluate more closely the technical and pharmacological issues while interpreting the results. The technical and pharmacological issues include the use of elastomeric pump balloons, which are sensitive to pressure, temperature, season, and patient activity, causing variability in infusion pump speed and resulting in variability in steady-state plasma concentrations of 5-FU (85, 86). Variability in infusion pump speed will also occur when using portable pumps, which essentially deliver a series of boluses. Due to the fact that even trace levels of DPD are present in blood, particularly the buffy coat, which makes 5-FU unstable after sample collection (87–90), it is crucial to properly separate the plasma and/or add DPD inhibitors. A sampling time of five half-lives following the start of the infusion does not yet equate to a sample at genuine steady state since, biologically, the elimination of 5-FU seems to fluctuate upon dosing. In fact, reaching steady-state 5-FU levels could take several hours (91–93). Furthermore, as DPD activity and maybe that of other 5-FU metabolizing enzymes exhibit some circadian rhythm (94, 95), variations in the timing of samples may further contribute to variability 84–88.

Nevertheless, it was unanimously agreed that by using the existing BSA-based dosing techniques and administering 5-FU by prolonged infusion, the interindividual variability in 5-FU plasma concentrations reached 40% and intraindividual variability reached 20%. Causes of interindividual variability include significant variation in DPD-mediated catabolism due to pharmacogenetic factors like SNPs in DPYD gene encoding DPD activity and environmental factors like nutritional status (68, 76).

Discounting technical and pharmacological issues, the intraindividual variability is considered to be modest and less than interindividual variability but significant enough for disproportionate systemic exposure (69) ^[22]^.

There are some important metrics of exposure that can correlate the clinical outcome, including AUC, time above threshold concentration, and C_max_ (73). The exposure to the drug (AUC) over the dosing interval is the most important PK correlate from a TDM perspective. The cumulative dosage and the exposure AUC are two metrics that have been shown to be substantially linked to clinical outcomes. Determining AUC for bolus 5-FU dosages is logistically challenging given the amount of samples that must be obtained in a short period of time. AUC measurements with 5-FU infusion schedules are relatively easier as only one sample is necessary with advanced modeling techniques, which is usually collected at steady state to predict the exposure (AUC) (84). The current consensus is a single sample at 18 h after the commencement of the infusion is the best parameter of evaluation, and the target therapeutic window is 20–30 mg/h/L (96).

### Evolution of TDM of 5-FU

In 1989, a study done by Santini et al. is the first study of TDM for 5-FU (71). In that PK study, AUC was measured and considered as an important PK parameter for predicting toxicity. AUC was used as a significant parameter for dosing in the second half of the cycle. The monitoring of these individuals’ PK has proven to be a reliable way to objectively improve the therapeutic index. AUC is reported as a measure of exposure in the majority of studies while Css is only rarely reported in earlier studies.

### Evidence for the exposure–response relationship

Using the existing BSA-based dosing techniques, there is about a 40% interindividual variability in 5-FU plasma concentrations *via* infusion. There is about 20% intraindividual variability in 5-FU plasma concentrations. Every drug has an exposure–response relationship that can be observed when comparing proximal biochemical effects brought on by target modification to drug concentration at the target site. Therefore, before attempting to change dose based on measures of such exposure, it is crucial to assess the exposure–response relationship.

Hillcoat et al. found a strong relationship between 5-FU plasma concentrations and tumor response in patients with gastrointestinal malignancies. In this study, patients received a 5-day continuous infusion of 5-FU given every 6 weeks at a dose of 1,200 mg/m^2^/day on days 1–5. The patients’ plasma 5-FU concentrations were estimated and found to differ greatly. Furthermore, plasma 5-FU area under the plasma concentrations × time curve (AUC) was shown to be considerably larger in patients with either a partial response (PR) or stable disease (SD) compared to individuals who did not have a tumor response. This was the first evidence of clinical data correlating 5-FU plasma exposure to clinical activity (72).

Martin et al. demonstrated the efficacy of therapeutic drug management (TDM) in individualized 5-FU dose in patients with metastatic colorectal cancer in routine clinical practice. Study results suggested that there was a decreased incidence of 5-FU-related toxicities and a much improved 5-FU exposure if TDM is adopted in clinical practice (90).

Yang et al. (2016) performed a meta-analysis to assess the efficacy and possible side effects of 5-FU using PK-guided vs. BSA-based dose modification in advanced malignancies using data from two randomized control trials (RCTs) and three observational studies involving 654 patients. A significant improvement in overall response rate (odds ratio = 2.04) compared with the conventional BSA technique was found to be an outcome of PK-monitored 5-FU therapy. The study revealed that PK-monitored 5-FU dosage has the potential to be used in colorectal cancer personalized therapy. The researchers concluded that PK-based 5-FU dosage showed superior overall response rate when compared to BSA-based dosing and improved toxicities irrespective of significant difference (77).

Fang et al. (2016) conducted a meta-analysis to evaluate the PKG-based algorithm for 5-FU compared with BSA-based methodology (5-FU). There were four studies (*n* = 504) included. The objective response rate to 5-FU treatment was “substantially” higher with the PKG algorithm than with the BSA-based method. Additionally, PKG was shown to “significantly” lower the probability of grade 3/4 potential adverse drug events (70). Salamone et al. (2017) conducted a study to validate the use of TDM to adjust 5-FU dose in mCRC patients under regular clinical settings. A total of 75 patients with metastatic colorectal cancer (mCRC) from eight German medical facilities participated in this trial. They were given up to six administrations of 5-FU infusions using the AIO (*n* = 16), FOLFOX6 (*n* = 26), or FUFOX (*n* = 33) regimens based on BSA. The subsequent 5-FU infusion dosages were modified in accordance with the 5-FU AUC of the preceding cycle in order to reach the desired AUC of 20 to 30 mg·h/L. The main goal was to demonstrate that TDM of 5-FU enhanced the proportion of patients in the desired AUC range at the fourth treatment compared to the first dose. Average 5-FU AUC at the time of the first administration was 18 + 6 mg·h/L, with 64%, 33%, and 3% of the patients having AUCs that were below, within, or beyond the target range, respectively. By the fourth dosage, 54% of patients were within the desired 5-FU AUC range (*p* = 0.0294), and the average 5-FU AUC was 25 + 7 mg·h/L (*p* < 0.001). Even though 55% of patients had their doses raised, the rates of grade 3–4 diarrhea (4.6%), nausea (3.4%), fatigue (0.0%), and mucositis (0.2%) associated with 5-FU were less compared to the historical data (97).

### Evidence for the exposure–adverse event relationship

A PK/PD study by Thyss determined the plasma concentration of 5-FU in 29 patients with head and neck cancer who were receiving a combination chemotherapy regimen that included cisplatin at a dose of 100 mg/m^2^ on day 1 and a continuous 5-day infusion of 5-FU at a dose of 1,000 mg/m^2^/24 h on days 2–6. In this study, a strong relationship was found between the incidence of toxicities such myelosuppression, mucositis, and diarrhea and greater systemic exposure to 5-FU (5-FU AUC > 30 mg·h/L) (98).

Twenty-one patients with advanced malignancies underwent 5-FU PK analysis by van Groeningen et al. from the Free University in Amsterdam, the Netherlands (99). 5-FU was given as an i.v. bolus once a week at a starting dose of 500 mg/m^2^, increasing the dose by 20% every 4 weeks until dose-limiting toxicity was noticed. The prevalence of toxicities was found to be strongly linked to 5-FU versus AUC using the logistic regression approach (96).

The first significant, prospective, multicenter, phase III randomized trial was carried out by Gamelin and colleagues in patients with mCRC receiving a weekly, 8-h infusion of 5-FU at a dose of 1,500 mg/m^2^. These patients would have been more likely to experience toxicity if the 5-FU dose had not been altered (100). This work is significant because it established for the first time that PK-guided 5-FU dose adjustment for the treatment of mCRC patients is practicable in clinical practice on an individual basis. The best way to ensure the right dose intensity for better results while limiting toxicity appears to be to evaluate plasma 5-FU AUC values. This method is more efficient and safe than dose adjustment based solely on clinical evaluation (100).

Yoshida and colleagues evaluated if there might be a relationship between the amount of 5-FU administered and the level of toxicity in 19 patients with mCRC. Patients in this study received a continuous i.v. infusion of 5-FU for 7 days straight at a dosage of 190–600 mg/m^2^/day. Nine patients (toxic group) out of the total study participants experienced toxic dermatitis, anorexia, nausea/vomiting, and >grade 2 stomatitis. AUC from 0 to 72 h after the start of 5-FU infusion (AUC_0–72 h_) and steady-state concentrations (CSS) of 5-FU were calculated. Between the individuals who experienced toxicity (*n* = 9) and those who did not (*n* = 10), there was a roughly twofold difference in the serum 5-FU CSS, AUC_72 h_, and total body clearance (101).

Deng et al. (2020) studied the efficacy of PK-based 5-FU dosing. A total of 153 patients with advanced colorectal cancer were randomly assigned to undergo either 5-FU chemotherapy with BSA-guided dose or a double-week chemotherapy regimen with 5-FU using PK dosing. When using AUC-based dosage, oral mucositis incidence dropped and the frequency of diarrhea was dramatically reduced. The researchers found that for patients with advanced CRC, PK-based dose control of 5-FU lowers the toxicity of chemotherapy and increases its long-term efficacy when compared to BSA-based dosing (102).

A randomized multicentric trial was conducted by Fety et al. (1998) to assess the clinical value of 5-FU dose adaptation guided by PK. A total of 122 patients with head and neck cancer were randomized to receive either the normal dose of cisplatin (100 mg/m^2^, day 1) and 5-FU (96-h continuous infusion) or a dose adjusted for the 5-FU level (AUC_0–48 h_; PKarm). A total of 106 patients were evaluated for toxicity and response. In comparison to the St-arm (*n* = 57), the 5-FU dosages and AUC were considerably lower in the PK-arm (*n* = 49) during cycles 2 and 3. Grade 3–4 neutropenia and thrombopenia were substantially more common in the St-arm compared to the PK-arm. In both treatment arms, the objective response rate was similar: 77.2% in the St-arm and 81.7% in the PK-arm. The authors concluded that therapeutic index can be improved by PK-based individual dose adaptation for 5-FU (103).

### Evidence for usefulness of TDM for 5-FU

Pharmacoeconomic considerations of cancer therapy become crucial while choosing a chemotherapeutic regimen. When patients frequently cannot receive therapy because of considerable financial burden labeled as “financial toxicity,” cost-effectiveness has now become an even more crucial factor to take into account. There are two studies [conducted by Goldstein (2014) and Soh (2015)] that evaluated the cost-effectiveness of TDM, and it has been observed that 5-FU TDM is economical in the management of both mCRC and SCCHN (104, 105). Associated toxicities with 5-FU, such as febrile neutropenia, nausea/vomiting, and diarrhea, can have serious negative clinical and cost effects on patients and the healthcare system. It is possible to modify the dosage of 5-FU depending on the quantity of 5-FU in the plasma, and it is obvious that PK-based dosing can greatly enhance clinical outcomes by lowering toxicities and boosting efficacy (104). In the UK, patients with mCRC treated with various 5-FU combination regimens had the potential cost benefit of PK-based versus BSA dosing of 5-FU examined (106). The cost-effectiveness of administering 5-FU by PK versus BSA in various standard chemotherapy regimens in the UK population was counterfactually simulated using a decision tree model, and all patients were presumptively treated with first-line therapy for 6 or 12 cycles or until disease progression. From the viewpoint of the national health system, the model’s costs were calculated, and it was found to be effective (106).

The use of 5-FU TDM in patients with mCRC resulted in significant cost savings and a gain in quality-adjusted life-years (QALYs), according to emerging data. 5-FU TDM should be regarded as a clinically significant and essential component of individualized therapy in the routine care of cancer patients in this era of precision medicine (73).

### Analytical methods employed for TDM of 5-FU

Compared to the majority of other anticancer medicines, 5-FU TDM faces obstacles that start much earlier in the preanalytical stage. Due to the widespread presence of the catabolic enzyme (DPD), which quickly converts 5-FU to its metabolite dihydro-5-FU, 5-FU is incredibly unstable in whole blood and plasma at ambient temperature. In order to separate plasma from cells, blood samples should typically be promptly put on ice and plasma extracted (73).

The last 40 years have seen huge breakthroughs in 5-FU TDM, and today, there is a validated algorithm of 5-FU dose adjustment based on plasma 5-FU levels to decrease toxicity and improve 5-FU efficacy. The levels of 5-FU in peripheral blood can be measured directly using a number of techniques, such as HPLC, GC-MS, and LC-MS/MS. Recently, a sensitive and accurate immunoassay for measuring 5-FU has become available. Comparing this test to conventional HPLC and LC-MS/MS procedures reveals significant logistical benefits. The immunoassay has a number of potential advantages over conventional HPLC and/or LCMS assays, including (1) a quicker turnaround time for clinical samples, (2) a smaller sample size requirement, and (3) automated quantitation using a reputable, validated clinical chemistry analyzer that can test a large number of samples at once. Conventional chromatographic techniques need sophisticated equipment and a higher level of staff training to operate the equipment. Given the necessity of more sample preparation stages and the lengthier time required to analyze samples and all calibrators before the sample result can be completed than immunoassay, the turnaround time for HPLC and/or LC-MS/MS procedures is often longer than immunoassay. Cross-reactivity of the antibody to analytes structurally related to the target analyte has historically been one of the possible drawbacks of immunoassays (107).

### Best practices for TDM of 5-FU

There are a number of crucial factors that must be taken into account while performing TDM 5 FU. Since 5-FU has a short half-life of only 10–15 min, steady-state conditions are expected 1 h after 5-FU infusion begins. Clinical studies actually suggest getting TDM samples at least 18 h following the onset of a 5-FU infusion (73, 91–93, 108). In view of this, the majority of the current protocols for 5-FU TDM suggest sampling be done on day 2 of a 48-h 5-FU infusion. Regarding the latter, blood sample is not advised if the infusion pump is empty or if it is thought to be within 30 min of emptying. Patients should be called back to the facility around 4 h before the expected completion of the drug infusion if blood collection for TDM is planned in order to prevent a significant fraction of TDM failures due to empty drug pumps. For patients undergoing 5-FU TDM, electric pumps are preferred over elastomeric pumps because they provide better timing accuracy, as the balloons of elastomeric pumps are sensitive to pressure, temperature, season, and patient activity (73, 85, 86). The blood sampled should be collected from a peripheral vein at a distance from the central port being used for 5-FU infusion. 5-FU is unstable in whole blood at room temperature due to *ex vivo* catabolism by DPD in the RBC; therefore, the collected blood sample should be placed on ice and plasma should be separated as soon as possible. Alternatively, a DPD inhibitor (gimeracil) may be added to the sample that allows centrifugation up to 24 h. However, a minimal turnaround time is important to continue the dose modification by modifying the infusion rate.

### Clinical impact of 5-FU TDM for precision dosing strategy

The conventional 5-FU dose based on BSA produces a wide range of 5-FU systemic exposure that correlates with a wide range of effectiveness measurements. BSA-based dosing allows for more customized 5-FU administration, although in adults, BSA does not correspond well with any PK measures (67). Many US centers use PK-based dosing to achieve a target concentration, and there are solid comparative data about the mortality benefit of TDM-guided 5-FU dose ^[32]^. By lowering toxicities and boosting efficacy, dose modification of 5-FU is doable and can greatly enhance therapeutic outcomes (90). Two prominent dose modification algorithms have been proposed. Gamerlin et al. recommended dose adjustment over a 5-FU concentration range on <4 to >31 mg·h/L (91) while the Kaldate algorithm is proposed for a wider range of concentration; 8–10 to >40 mg·h/L (108). The proposed dose modification algorithm by Kaldate has been prospectively validated in a single cohort of patients in a clinical trial setting (90) and hence also the algorithm of choice as recommended by the IATDMCT consensus statement for TDM of 5-FU in oncological practices (73). Bayesian forecasting based on PB-PK modeling and simulation is an upcoming strategy proposed for MPID of 5-FU. The greatest advantage it may offer is to do away with the DPD genotyping or phenotyping for dose optimization of 5-FU. The MPID has been proposed to overcome genotype or phenotypic misclassification, by predicting the clearance of the drug accurately (109).

## Methotrexate

Mtx is an antifolate drug used in low doses (7.5–25 mg/week) as a disease-modifying antirheumatic drug (DMRD) whereas it is used at large doses (i.e., >500 mg/m^2^ intravenously) to treat a variety of malignancies [(acute lymphoblastic leukemia (ALL), non-Hodgkin lymphoma (NHL), osteosarcoma, and medulloblastoma]. The majority (90%) of Mtx is cleared unchanged *via* the kidneys, with the hepatic metabolism producing only a minor amount of metabolite 7-hydroxy methotrexate. The systemic clearance is roughly 50–135 ml/min/m^2^, and the terminal half-life ranges from 8 to 15 h. The PK, pharmacodynamic, and toxicity profiles depend on the dose (110). Upon entering the cells, Mtx is converted into a series of Mtx-polyglutamates (Mtx-PGs). High-dose Mtx (HDMtx) acts through inhibition of the dihydrofolate reductase (DHFR) enzyme with very high potency and hence blocks the purine synthesis in the s-phase of the cell cycle, thereby preventing cell proliferation in both tumor and normal cells. At low doses, Mtx-PGs prevent the inflammatory processes in the white blood cells and hence prevent the immune-mediated damage. Both low-dose and high-dose Mtx may cause life-threatening events such as hepatotoxicity, myelosuppression, and pulmonary toxicity, but the high-dose therapy may cause potentially life-threatening nephrotoxicity due to crystallization of the drug in the nephrons, which is rarely seen with low-dose therapy (111). Therefore, dose reduction is recommended when the creatinine clearance is decreased (112). The guiding principle is maintaining hydration, urinary alkalinization, monitoring serum creatinine, and a pharmacokinetically guided leucovorin rescue with TDM.

### Rationale for TDM of methotrexate

HDMtx therapy meets the most important criteria for TDM, a large inter- and intraindividual variability in the systemic concentration achieved (113–115). The extent of interpatient variability is 52% and intrapatient variability is as high as 48% (116). This is reflected in the wide range of incidence of nephrotoxicity from 1.8% to 10.7% (110, 117). Pharmacogenetic factors like SNPs of MRP2 and OATP1B1 affecting the expression of these transport proteins partly contribute to the interindividual variability of disposition of Mtx. Several drug–drug interactions (NSAIDs, ciprofloxacin, probenecid, sulfamethoxazole, trimethoprim, amiodarone, tyrosine kinase inhibitors, and proton pump inhibitors) and known disease conditions like Down’s syndrome. Coupled with all these known factors that affect Mtx elimination, there is an unexplained and variable delay in the renal clearance of Mtx despite all preventive measures taken. Maintaining the concentration in a nontoxic and efficient target range is made possible by using TDM and optimizing the doses of MTX and leucovorin. HDMtx is one of the prototype oncologic scenarios for which TDM had been employed (118).

### Evidence for the concentration–toxicity relationship

Classically, the purpose of Mtx TDM has been to monitor the plasma concentration of the drug to avoid toxicity. Most lines of evidence come from observational studies and not from the randomized controlled clinical trials for obvious reasons. The plasma levels that define toxic exposure evolved over time. In one of the earliest studies by Stoller et al., it was suggested that a concentration >0.9 μM/ml at 48-h increases the risk of myelotoxicity (119, 120). These levels are important to guide the dose of folinic rescue therapy. Pediatric ALL consensus statement recommends a standard 50 mg/m^2^ infusion of leucovorin every 6 h, if the Mtx concentration is >20 μM/ml at 36 h, >10 μM/ml at 42 h, and >5 μM/ml at 48 h. Supplemental leucovorin must be administered until the Mtx level falls to 0.1 to 0.05 μM/ml. A delay in measurement of plasma Mtx concentration by 24–36 h equates to having an uncompromised therapeutic action of the drug. It would be interesting to note that while Mtx nephrotoxicity has been most consistently associated with plasma concentration across studies, it has been found to be a poor predictor of other toxicities like myelotoxicity and hepatotoxicity with variable results reported from studies (118). If acute kidney injury occurs with high-dose Mtx therapy, or an extremely high concentration of Mtx is found in the blood, glucapidase (carboxypeptidase G2) is the drug of choice, and not leucovorin, as it is extremely efficient and has removed 98% of the Mtx in first 30 min.

### Evidence for the concentration–response relationship

Mtx has long been used as a disease-modifying agent in low doses (<50 mg/m^2^) in many rheumatic and autoimmune diseases. Due to its low cost, it is still the drug of choice in such chronic diseases (121, 122). However, 40% of the patients do not show clinical response to Mtx. Some of them have early nonresponse and some cease to respond after an initial period of nonresponse. Apart from the reasons like noncompliance and inadequate dosing, the variation in Mtx uptake and metabolism has been postulated to play a role. The cellular uptake of Mtx and their polyglutamation rate may be important contributors for such variable response. The activity of the folypolyglutamase synthase enzyme, which is responsible for polyglutamation, and the levels of Mtx-PGs have been proposed to be the biomarkers for detecting patients who are at the risk of nonresponse (123). Sensitive LC-MS/MS-based methods have been developed to detect them with precision. Studies have shown that there is a great variation in the levels of polyglutamates in the blood cells for patients receiving the same dose of Mtx (123). De Rotte et al. have proposed a multivariate model including age, gender, folate status, and genotype to correlate the disease activity with the polyglutamate levels (124). Recently, a randomized controlled study showed that daily therapy is as effective as weekly therapy when the polyglutamate-3 levels are similar. This study also showed that obese patients achieve a lower concentration of Mtx-PG3 (125). Clinical response in several other diseases like juvenile rheumatoid arthritis, juvenile dermatomyositis, and inflammatory bowel disease was also reported to be affected by the Mtx-PG concentrations. However, this is still investigated and not yet ready for clinical use; more concrete evidence is needed.

### Best practices for TDM of Mtx

For the 24-h infusion schedule, it is advised to use TDM at least 24 h (the end of the infusion), 48 h, and 72 h following the start of high-dose MTX infusion, until the concentration is below 0.1–0.2 mol/L. The TDM protocols can be divided into two modes: The steady-state concentration for the 24-h infusion regimen is thought to be C24h (the end of infusion), which is correlated to efficacy and safety, whereas C48h and C72h are mostly related to safety. C3-6h (the end of the infusion), for the rapid infusion (less than 6 h) regimen, is thought to represent the peak concentration in terms of efficacy and safety, while C24h, C48h, and C72h are primarily connected to safety. In most circumstances, the safe MTX concentration range is below 0.1–0.2 mol/L, and TDM can be discontinued once it is attained. It is important to note that MTX levels less than 0.05 mol/L can be regarded as a stricter safe range for patients with delayed MTX elimination and/or indications of acute renal dysfunction.

### Analytical methods employed for TDM

Both MTX and its metabolites can be distinguished and detected using the HPLC and HPLC-MS/MS procedures, although they are expensive and require sophisticated sample processing. Automated immunoassay platforms are also available for Mtx concentration measurement. Both liquid chromatography and immunoassay could be used in a TDM laboratory. It must be kept in mind that the immunoassays have the disadvantage of slightly overestimating the concentrations due to nonspecific interactions with the Mtx metabolites. Another instance where immunoassay can falter is during treatment with glucarpidase, as Mtx degradation products interfere with the analysis and true estimates may not be found. Therefore, chromatographic methods must be employed during this situation.

### Clinical impact of TDM in Mtx dose optimization

Apart from the pharmacokinetically dosing leucovorin, Mtx TDM data have also been utilized to build dose prediction models taking into account covariates like creatinine clearance and alanine transaminase levels to forecast the dose. This is to facilitate a personalized dose for the patients, which attempts to attain the desired fall in the concentration of Mtx in a desired time frame and minimizing hospital stay (126, 127). These studies reported the lower incidence of nephrotoxicity, the therapeutic concentration of Mtx was within the therapeutic range, and the clearance of Mtx was as anticipated (94, 95).

The main purpose of using HDMtx administration in patients with ALL and NHL is to address the CNS disease and to reduce risk of CNS relapse. Most studies with Mtx TDM have been performed on serum samples. A study on 138 CSF samples from children with ALL and NHL was conducted to evaluate the Mtx concentration in the cerebrospinal fluid (CSF). Serum Mtx concentrations at the end of infusion were assessed by routine TDM. Cytotoxic Mtx concentrations of 1 μM or greater were detected in 81.2% of CSF samples before administering intrathecal Mtx. One micromolar concentration is proposed to provide the desired antileukemic effect to prevent CNS relapse. This important study made a case for reducing the need for intrathecal injection of Mtx and, hence, reducing the risk of multiple lumbar puncture in children suffering from ALL and NHL (128).

## Future directions

Bayesian modeling and simulation-based MIPD are some of the noteworthy precision medicine tools likely to be implemented in routine patient care in the near future. Busulfan has made a significant progress in terms of clinical testing for MIPD, though widespread application of Bayesian adaptive dosing is yet to be achieved. For 5-FU, it is still in the evolution phase and a through work in this direction is needed. For Mtx, two extremes of doses are practiced; thus, this calls for an Mtx TDM separately for autoimmune diseases apart from the well-established practice for HDMtx. Utilizing the chronic toxicity data of Mtx TDM could be tuned in for understanding the Mtx nonresponder profile in patients with rheumatic diseases. Though a few model informed dosing are in practice, it is time to refine these strategies and the development of user-friendly clinical decision support tools and their wider application. The reduction in the turnaround time in TDM reporting along with the implementation of MIPD should be aimed for in the future to make quicker as well as smarter therapeutic decisions in patient care. In addition, pharmacometabolomic approaches are gaining momentum and their association with different clinical end-points have also been demonstrated (129, 130).

## Author contributions

PS accepted the invitation and built the team. She conceived the idea, compiled the first draft with PA and JK. PG provided the clinical inputs and practice oriented points. All authors contributed to the article and approved the submitted version.
